# Surgical accuracy, function, and quality of life of simultaneous versus staged bilateral Total hip Arthroplasty in patients with Osteonecrosis of the femoral head

**DOI:** 10.1186/s12891-017-1605-2

**Published:** 2017-06-17

**Authors:** Seung-Chan Kim, Young-Wook Lim, Woo-Lam Jo, Dong-Chul Park, Jin-Woo Lee, Won-Woo Kang, Yong-Sik Kim

**Affiliations:** Department of Orthopaedic Surgery, Seoul St. Mary’s Hospital, College of Medicine, The Catholic University of Korea, Banpodae-ro 222, Seocho-gu, Seoul, 137-701 South Korea

**Keywords:** Total hip arthroplasty, Bilateral, Simultaneous, Staged, Accuracy, Osteonecrosis of the femoral head

## Abstract

**Background:**

The optimal surgical option for patients requiring bilateral hip replacement remains controversial. The purpose of this study was to compare surgical accuracy; functional outcome and health-related quality of life; and prosthetic-related complications and revision surgery of a simultaneous bilateral total hip arthroplasty (THA) with those of a staged bilateral THA with an interval between procedures <12 months.

**Methods:**

A total of 123 unselected consecutive patients (mean age, 43.3 years) who underwent bilateral THAs for osteonecrosis of the femoral head (ONFH) with a minimum follow-up of two years (mean, 60.2 months) were studied retrospectively; 63 simultaneous procedures served as a test group and 60 staged procedures served as a control group.

**Results:**

The mean postoperative leg-length discrepancy (LLD) and the percentage of patients who had an LLD >3 mm were significantly lower in the simultaneous group (*P* < 0.001 and *P* = 0.001, respectively). A higher number of cups within the safe zones, a higher correction rate, and a lower failure rate for the cup placement in the second-operated hip were also identified in the simultaneous group. The mean Harris hip score, EuroQol-5D index, and EuroQol-visual analogue scale score were all better in the simultaneous group at the latest follow-up (*P* < 0.001, in all comparisons). We found that the simultaneous procedure was associated with a lower incidence of postoperative prosthetic-related complications and revision surgery.

**Conclusions:**

We suggest that bilateral ONFH could be treated with a simultaneous THA rather than a staged THA to achieve a better surgical outcome.

## Background

It has been estimated that approximately 15–25% of patients being considered for primary total hip arthroplasty (THA) require a second contralateral procedure within five years [1–3]. Specifically, non-traumatic osteonecrosis of the femoral head (ONFH) is a progressive disease primarily affecting bilateral hip joints of young adults [4, 5]. Once ONFH begins, 80% of femoral heads may collapse if no treatment is administered, thus, it frequently may result in the need for THA [6]. However, the optimal timing of surgery in patients requiring bilateral hip replacement still remains controversial, and the surgeon and patient must decide whether to perform simultaneous bilateral THA (BTHA) or staged BTHA with an interval between procedures.

Simultaneous BTHA has several potential benefits over a staged procedure, including a decrease in cost and overall length of hospital stay, use of a single anesthetic, better functional recovery, and earlier return to daily activities [7, 8]. Simultaneous BTHA, however, also poses a potential increased risk of venous thromboembolic events, heterotopic ossification, higher blood transfusion requirement, and increased need for transfer to a rehabilitation facility [2, 3, 8–13]. Nevertheless, there is a recent consensus that there is no significant difference in the safety of the simultaneous BTHA and the staged BTHA in regard to complication rate and mortality [8, 9, 14, 15]. However, some studies have not supported this practice [2].

Although the literature contains a number of studies regarding perioperative and socioeconomic implications of simultaneous BTHA vs. staged BTHA, to the best of our knowledge, only a few studies have compared accuracy in surgical procedures, radiographic outcome, functional outcome, and quality of life. We hypothesized that patients who underwent a simultaneous BTHA would have superior surgical accuracy, better functional outcome, and quality of life. We secondarily hypothesized that there would be no difference in surgical outcome of the first-operated hip of a BTHA between the two procedures.

Therefore, we sought to (1) compare the postoperative leg-length discrepancy (LLD) and the accuracy of acetabular cup placement (in particular, of the second-operated hip) based on the safe zones proposed by Lewinnek et al. [16] and Callanan et al. [17]; (2) compare the functional outcome and quality of life in patients with simultaneous BTHA versus those in patients with staged BTHA; and (3) determine whether differences in the incidence of perioperative prosthetic-related complications and revision surgery were present between the two groups.

## Methods

After institutional review board approval, we retrospectively reviewed a consecutive series of 177 patients (354 hips) who underwent bilateral cementless THAs from October 2007 through October 2013 with either a simultaneous or staged procedure. All patients with a preoperative diagnosis of bilateral ONFH and known clinical outcomes through regular follow-up (minimum follow-up period: two years) were considered for inclusion. Patients with primary or secondary osteoarthritis, inflammatory arthritis, bony ankylosis, neurologic diseases, previous hip surgery, and significant involvement of the knee, ankle and/or spine were excluded. Ultimately, a total of 123 patients (246 hips), consisting of 71 male (142 hips) and 52 female patients (104 hips), were included in the study. Mean patient age at the time of surgery was 43.3 years (range, 17 to 77) and mean follow-up duration was 60.2 months (range, 24 to 101).

The patients were divided into two groups according to type of surgery; 63 patients who underwent simultaneous BTHA, comprised the test group; the control group comprised 60 patients whose operations were performed as a staged procedure with an interval < 12 months (mean, 4.8 months; range, 2 weeks to 12 months). The procedure was selected primarily based on symptoms and patient preference. A simultaneous procedure was chosen if the patient presented with bilateral intractable hip pain, while a staged procedure was planned when the patient presented with one-sided intractable pain or preferred to undergo a two-stage procedure. A summary of the demographics and clinical data of the patients of the two groups is shown in Table 1. No significant differences were found between the two groups in regard to age, sex, body mass index (BMI), American Society of Anesthesiologists (ASA) grade, disease stage, ceramic head diameter implanted, follow-up duration, operative time, and intraoperative blood loss; however, a shorter length of hospital stay and a smaller amount of hospital charge were noted in the simultaneous BTHA group.

**Table 1:** Patient demographics and clinical data

Data	Simultaneous BTHA	Staged BTHA	*P* value
Number of patients (hips)	63 (126)	60 (120)	
Mean age (years; range)	43.1 (20 to 69)	43.5 (17 to 77)	0.871^#^
Sex (n; %)			0.336^†^
Male	39 (62)	32 (53)	
Female	24 (38)	28 (47)	
Mean BMI (kg/m^2^; range)	22.9 (15.8 to 30.9)	23.3 (16.4 to 37.9)	0.568^#^
ASA grade (n; %)			0.404^†^
1	19 (30)	18 (30)	
2	38 (60)	40 (67)	
3	6 (10)	2 (3)	
4/5	0	0	
ARCO stage in ONFH (hips; %)			0.638^†^
III	89 (71)	88 (73)	
IV	37 (29)	32 (27)	
Ceramic head (n; %)			0.817^†^
28 mm	18 (14)	14 (12)	
32 mm	40 (32)	38 (31)	
36 mm	68 (54)	68 (57)	
Mean follow-up (months; range)	57.8 (24 to 98)	62.7 (24 to 101)	0.767^‡^
Mean length of hospital stay (days; range)	10.5 (4 to 28)	18.7 (8 to 45)	< 0.001^**‡**^
Mean hospital charge ($; range)	12,608 (9645 to 21,469)	14,910 (11,428 to 27,146)	< 0.001^‡^
Mean operative time (min; range)	172 (108 to 245)	162 (104 to 275)	0.058^#^
Mean intraoperative blood loss (mL; range)	1037 (550 to 2000)	1145 (600 to 2700)	0.098^‡^

*BTHA* bilateral total hip arthroplasty, *ASA* American Society of Anesthesiologists, *ONFH* osteonecrosis of the femoral head, *ARCO* Association Research Circulation Osseous

^#^Independent *t*-test

^†^Chi-square test

^‡^Mann-Whitney test

In all patients, preoperative planning with digital templating on standardized radiographs of the pelvis was performed. Preoperative templating was performed to determine the hip center of rotation, femoral offset, and preoperative LLD, as well as to estimate the optimal size and position of the acetabular and femoral component. The radiographs comprised an anteroposterior (AP) view of the pelvis centered over the pubic symphysis with the pelvis and legs in a neutral position and at 15° of internal rotation to control for femoral anteversion, and lateral views of both hips. Each template radiograph included a standard calibration marker positioned as close to the coronal plane of the hip joint as possible; thus, facilitating the adjustment of the magnification. The head-to-lesser-trochanter distance (HLD) was measured as described in our previous study and recorded preoperatively as a reference standard for minimizing the occurrence of LLD in all cases [18]. In our unit, all radiographs are digitized using the Picture Archiving and Communication System (PACS; Marosis, Infinite, Seoul, Korea).

**Fig. 1:**
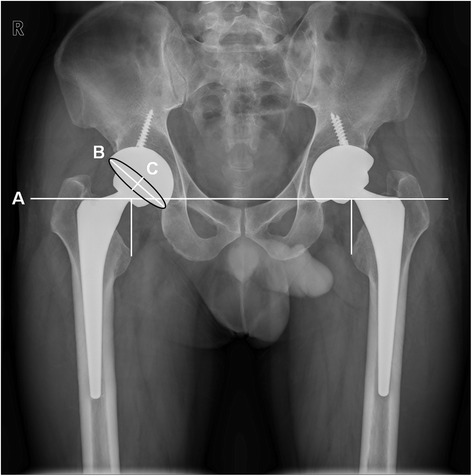
A standardized anteroposterior pelvic radiograph of a 39-year-old male patient with bilateral osteonecrosis of the femoral head at 6 months after simultaneous bilateral total hip arthroplasty showing the radiographic measurement of leg-length discrepancy and acetabular cup orientation. The line A indicates the inter-teardrop line as a reference line. The line B is the long axis and the line C is the short axis of the opening ellipse of the acetabular component, respectively

Six months postoperatively, standardized pelvic radiographs were taken on the radiographic coronal plane to evaluate the postoperative LLD, radiographic cup inclination and anteversion angle the same as the protocol for preoperative AP pelvic radiographs (Fig. 1). When taking standardized plain radiographs, effort was made to decrease an anterior or posterior pelvic tilt throughout the study. The LLD on radiographs was defined as the difference in perpendicular distance in millimeters between the inter-teardrop-line to the corresponding tip of the lesser trochanter [19]. The actual value of the LLD was obtained using a known size of the prosthetic head implanted. The orientation of acetabular components was measured with the use of digital measurement tools on PACS workstations. The cup inclination angle was directly measured as the angle between the long axis of the opening ellipse and the inter-teardrop line. The anteversion angle was computed using the method described by Lewinnek et al. (= arcsin (short axis/long axis)) [20, 21]. Thus, the angles measured on a plain AP radiographs by this method were radiographic anteversion and inclination since the radiographic definition and radiographic coronal plane (functional coronal plane) were consistently used in this study; Lu et al. [22] demonstrated that measurement of the orientation of acetabular components on radiographic coronal plane using Lewinnek’s method is reliable and accurate compared with measurement on CT scans. To test a cup alignment, two safe zones for inclination and anteversion (the safe zone of Lewinnek et al. [16] (inclination, 30° to 50°; anteversion, 5° to 25°) and the modified safe zone of Callanan et al. [17] (inclination, 30° to 45°; anteversion, 5° to 25°)) were used as standard references in this study. Calculation of the number of hips that were in those safe zones regarding inclination, anteversion, and a combination of both were done for both groups. For the analyses of surgical accuracy in positioning of the acetabular component, the cup placement of the second hip of the BTHA was determined as ‘correction’ if the cup of the second-operated hip was within the safe zone while the cup of the first-operated hip was outside the safe zone. In contrast, the cup placement of the second hip was determined as ‘failure’ if the cup of the second hip was outside the safe zone while the cup of the first hip was within the safe zone. One independent investigator (one of the authors) who was not involved in any of the operations and blinded to the study groups, evaluated all radiographs.

All surgeries were performed by a single surgeon (the senior author) at our institution under general anesthesia in a standard fashion. A posterolateral approach with a short external rotator preservation procedure was used to enhance joint stability with the patient in the lateral decubitus position for all procedures [23]. All patients received a cementless hemispheric porous-coated acetabular component (BENCOX^®^; Corentec, Cheonan, Korea) and a cementless double-tapered wedge femoral component (BENCOX^®^; Corentec) with ceramic-on-ceramic bearing couples (BIOLOX^®^; CeramTec AG, Polchingen, Germany). The surgeon positioned the cup using its aligning guide with the goal of cup position at 40° of abduction and 15° of anteversion. All THAs were performed with the use of the HLD method to minimize the occurrence of LLD [18]. Additionally, the legs were palpated on the table intraoperatively to confirm the leg length. In cases of simultaneous BTHA, the surgical team strove to reduce the time for repositioning the patient and preparing the skin of the second hip. Nevertheless, the average time from the skin closure of the first hip to the skin incision of the second hip was approximately 15 min (mean, 15.3 min). The operative time, intraoperative blood loss, and any intraoperative complications were recorded in the operative notes immediately after surgery.

Clinical and radiologic evaluations were performed at 6 weeks, 3, 6, 12 months, and yearly thereafter postoperatively. The patients were assessed with the Harris Hip Score (HHS) at each follow-up visit to evaluate functional outcome and completed the self-administered EuroQol-5 dimension (EQ-5D) questionnaires and the EuroQol-visual analogue scale (EQ-VAS) measurement at the time of the latest follow-up to evaluate health-related quality of life and health status [24]. The EQ-5D has five dimensions (mobility, self-care, usual activities, pain/discomfort, and anxiety/depression) and each item has three levels (no problem, some problem, or major problem); an EQ-5D index score of 1 represents the best possible health status, and a score of 0 represents the worst possible health status. The EQ-VAS is a vertical visual analogue scale ranging from 0 (indicating the worst imaginable health status) to 100 (indicating the best imaginable health status). The incidence and details of any postoperative prosthetic-related complications and revisions at any time point during follow-up were noted. Postoperative systemic complications were not addressed in this study except for death since those have been evaluated in several previous studies [2, 8, 9, 14, 15, 25–27]. All clinical information was collected by a research assistant (one of the authors) using medical records.

A post hoc power analysis was performed using a G*Power software [28] (version 3.1; Düsseldorf University, Düsseldorf, Germany) for each sample size of the cohorts and the means (and standard deviations) of the outcome measure LLD of each group, resulting in a power value of 0.98. Statistical analysis was performed using SPSS for Windows (version 21; SPSS, Chicago, Illinois, USA). The distribution of variables was tested for normality using the Kolmogorov-Smirnov test. The independent *t*-test was used for comparison of normally distributed continuous variables and the Mann-Whitney U test was used for nonparametric continuous variables in independent groups. The Pearson chi-square test or Fisher’s exact test was used for categorical variables. *P* < 0.05 was considered statistically significant.

## Results

Among 246 hips with ONFH, no significant difference was found between the simultaneous and staged BTHA groups in stage of the disease based on the Association Research Circulation Osseous (ARCO) classification system [29]. At the time of each surgery, there were 89 stage-III hips and 37 stage-IV hips in the simultaneous BTHA group, and 88 stage-III hips and 32 stage-IV hips in the staged BTHA group (*P* = 0.638). The independent comparison of the second-operated hips also showed no significant difference in stage between the two groups: 49 stage-III hips and 14 stage-IV hips in the simultaneous group, and 45 stage-III hips and 15 stage-IV hips in the staged group (*P* = 0.717).

The mean postoperative LLD was significantly lower in the simultaneous BTHA group (2.1 mm) compared to the staged BTHA group (4.3 mm; *P* < 0.001). A percentage of patients who had an LLD < 3 mm postoperatively in the simultaneous group was significantly higher than that of the staged group (75% vs. 42%; *P* = 0.001). We still found a higher percentage of patients in the simultaneous group who had an LLD < 5 mm, although significance of the difference disappeared (92% vs. 73%; *P* = 0.068). No patient (0%) had an LLD of >10 mm in the simultaneous group, whereas 2 patients (3.3%) had an LLD of >10 mm in the staged group (one with an LLD of 14.1 mm, and the other with an LLD of 10.8 mm).

There were no significant differences between groups in the mean radiographic cup inclination angle or anteversion angle for the first-operated hip as well as the second-operated hip of the BTHA (Table 2). When comparing the first-operated hip of the simultaneous group to that of the staged group independently, no significant differences in the number of cups within each safe zone for inclination and anteversion were found. However, the comparison of the second-operated hip showed significant differences between the two groups. The number of cups within each safe zone for inclination was found to be higher in the simultaneous group: 95% vs. 85% for Lewinnek (*P* = 0.056), and 81% vs. 65% for Callanan (*P* = 0.046). The number of cups within the safe zone for anteversion was also significantly higher in the simultaneous group: 95% vs. 77% (*P* = 0.003).

**Table 2:** Comparison of accuracy in acetabular cup placement according to type of surgery

Parameter	Simultaneous BTHA (*N* = 63)	Staged BTHA (*N* = 60)	*P* value
Mean inclination (°; mean ± SD; range)			
First-operated hip	42.0 ± 6.2 (29.3 to 55.7)	40.8 ± 7.3 (24.5 to 61.6)	0.333^#^
Second-operated hip	40.7 ± 5.4 (22.8 to 52.4)	41.7 ± 7.2 (25.8 to 63.5)	0.401^#^
Mean anteversion (°; mean ± SD; range)			
First-operated hip	16.7 ± 6.9 (2.7 to 30.5)	17.3 ± 7.5 (4.7 to 36.3)	0.639^#^
Second-operated hip	17.9 ± 5.2 (7.8 to 30.8)	17.5 ± 7.7 (3.8 to 34.6)	0.706^#^
Cups within safe zone for inclination (n; %)			
First-operated hip			
Lewinnek (30° to 50°)	56 (89)	51 (85)	0.522^†^
Callanan (30° to 45°)	42 (67)	45 (75)	0.310^†^
Second-operated hip			
Lewinnek (30° to 50°)	60 (95)	51 (85)	0.056^†^
Callanan (30° to 45°)	51 (81)	39 (65)	0.046^**†**^
Cups within safe zone for anteversion (n; %)			
First-operated hip			
Lewinnek, or Callanan (5° to 25°)	50 (79)	47 (78)	0.889^†^
Second-operated hip			
Lewinnek, or Callanan (5° to 25°)	60 (95)	46 (77)	0.003^†^

*BTHA* bilateral total hip arthroplasty, *SD* standard deviation

^#^Independent *t*-test

^†^Chi-square test.

In the analyses of accuracy of acetabular cup placement of the second-operated hip, the simultaneous group showed a higher correction rate and a lower failure rate for both inclination and anteversion (Table 3). The failure rate for anteversion in the simultaneous group, in particular, was significantly lower: 2% vs. 23% (*P* = 0.001).

**Table 3:** Comparison of correction rate and failure rate in acetabular cup placement during second THA according to type of surgery

Parameter	Simultaneous BTHA (*N* = 63)	Staged BTHA (*N* = 60)	*P* value
Correction rate for inclination (n; %)			
Lewinnek (30° to 50°)	7/7 (100)	7/9 (78)	0.475^‡^
Callanan (30° to 45°)	16/21 (76)	9/15 (60)	0.465^‡^
Failure rate for inclination (n; %)			
Lewinnek (30° to 50°)	3/56 (5)	7/51 (14)	0.188^‡^
Callanan (30° to 45°)	7/42 (17)	15/45 (33)	0.074^†^
Correction rate for anteversion (n; %)			
Lewinnek, or Callanan (5° to 25°)	11/13 (85)	10/13 (77)	1.000^‡^
Failure rate for anteversion (n; %)			
Lewinnek, or Callanan (5° to 25°)	1/50 (2)	11/47 (23)	0.001^†^

*BTHA* bilateral total hip arthroplasty

^†^Chi-square test

^‡^Fisher’s exact test.

The mean postoperative HHS, EQ-5D index, and EQ-VAS score at the time of the latest follow-up in the simultaneous group were all significantly higher than those of the staged group (Table 4).

**Table 4:** Comparison of postoperative Harris hip score, EQ-5D and EQ-VAS score at the time of the latest follow-up after THA according to type of surgery

Parameter	Simultaneous BTHA (*N* = 63)	Staged BTHA (*N* = 60)	*P* value
Harris hip score (mean ± SD; range)	95.9 ± 4.8 (73 to 100)	90.7 ± 8.2 (64 to 100)	< 0.001^**†**^
EQ-5D_index_ (mean ± SD; range)	0.94 ± 0.08 (0.72 to 1.00)	0.87 ± 0.09 (0.68 to 1.00)	< 0.001^†^
EQ-VAS (mean ± SD; range)	86.0 ± 10.6 (50 to 100)	74.1 ± 15.6 (30 to 100)	< 0.001^†^

*BTHA* bilateral total hip arthroplasty, *SD* standard deviation

^†^Mann-Whitney test.

The results of perioperative prosthetic-related complications and revision surgery in the two groups are summarized in Table 5. No dislocations occurred in either group and there were no significant differences in the overall incidence of intraoperative fracture, dislocation, aseptic loosening, wound infection, periprosthetic fracture, or revision between the two groups. However, both the number of occurrences in the second hip and the proportion of second hip occurrences in the staged group were higher than those in the simultaneous group for all events except dislocation and periprosthetic fracture. On the contrary, the proportion of second hip occurrences in the simultaneous group did not exceed 50% for all events. In the simultaneous group, there were 2 revisions (1.6%; 1 of 2 performed on the second hip) for aseptic loosening of the femoral component during the entire follow-up period. Four revisions (3.3%; 4 of 4 performed on the second hip) were required in the staged group; these included 1 femoral component revision and 1 acetabular component revision for aseptic loosening, and 2 two-stage revisions for septic loosening (one was on the acetabular component and the other on the femoral component). Mortality did not differ significantly between the two groups at any time point (Table 5).

**Table 5:** Prosthetic-related complications and mortality of simultaneous and staged BTHA during entire follow-up period

Complication	Simultaneous BTHA (126 hips)		Staged BTHA (120 hips)		*P* value
	Overall incidence	Proportion of second hip given occurrence (n; %)	Overall incidence	Proportion of second hip given occurrence (n; %)	
Intraoperative fracture (n; %)^a^	10 (8)	3/10 (30)	7 (6)	5/7 (71)	0.516^†^
Dislocation (n; %)^a^	0 (0)	0/0 (0)	0 (0)	0/0 (0)	1.000^‡^
Aseptic loosening (n; %)^a^					
Acetabular component	0 (0)	0/0 (0)	1 (1)	1/1 (100)	0.488^‡^
Femoral component	2 (2)	1/2 (50)	1 (1)	1/1 (100)	1.000^‡^
Superficial or deep wound infection (n; %)^a^	0 (0)	0/0 (0)	2 (2)	2/2 (100)	0.237^‡^
Periprosthetic fracture (n; %)^a^	0 (0)	0/0 (0)	0 (0)	0/0 (0)	1.000^‡^
Revision surgery (n; %)^a^	2 (2)	1/2 (50)	4 (3)	4/4 (100)	0.437^‡^
Mortality (n; %)^b^					
6 weeks	0 (0)	-	0 (0)	-	1.000^‡^
1 year	1 (2)	-	0 (0)	-	1.000^‡^
2 year	1 (2)	-	1 (2)	-	1.000^‡^

*BTHA* bilateral total hip arthroplasty

† Chi-square test

‡ Fisher’s exact test

^a^ Data are presented as number of joints affected

^b^ Data are presented as number of patients affected

## Discussion

Patients often present with bilateral hip disease, and the patient and surgeon must decide whether to perform simultaneous bilateral THA or unilateral THA, with replacement of the other hip in the future. Since the introduction of the simultaneous procedure in 1967 [30], the two procedures have been repeatedly evaluated in regard to safety and socioeconomic factors [2, 8, 25–27]. However, the literature contains only a limited amount of studies that assess the accuracy of the surgical procedure, radiographic outcome, functional outcome, and quality of life in regard to simultaneous versus staged BTHA. In patients with bilateral ONFH, the present study showed that simultaneous BTHA resulted in a superior surgical accuracy, better functional outcome, higher quality of life, and fewer prosthetic-related complications, compared to a staged BTHA. In particular, there has been a remarkable difference regarding accuracy of cup placement when comparing the second-operated hip independently between the two groups.

The restoration of normal anatomy and biomechanics after THA, such as leg-length and acetabular orientation, are thought to be important factors in achieving pain-free and well-functioning hip joint, as well as ensuring the stability of the prosthetic hip and patient satisfaction [18, 19, 31, 32]. There currently is no defined limit of acceptable LLD in terms of hip function and patient satisfaction. Nevertheless, there is a general consensus among hip surgeons that most patients can tolerate a discrepancy of <10 mm [33, 34]. We have found a significantly lower LLD after a simultaneous BTHA than that after a staged procedure. In the simultaneous group, no patient had an LLD of >10 mm, whereas 2 patients had an LLD of >10 mm in the staged group and had much lower clinical scores than the other patients (one with an LLD of 14.1 mm, HHS of 70, EQ-5D index of 0.757, EQ-VAS of 60; and one with an LLD of 10.8 mm, HHS of 65, EQ-5D index of 0.766, EQ-VAS of 50). Our findings do not agree with a previous study reporting no significant difference in the postoperative LLD (4.5 mm vs. 5.3 mm*; P* = 0.239) [9]. In their study, however, a lower LLD was also identified in the simultaneous group.

Our findings indicate that a simultaneous BTHA is associated with a superior surgical outcome. In this study, the second-operated hips in the simultaneous group showed better results in regard to the number of cups within the safe zones and the correction rate/failure rate for the acetabular cup placement, compared to those of the staged group. In a previous randomized controlled trial, Bhan et al. [9] found no significant difference in the mean abduction angle of the acetabular component between the two groups. However, unlike the present study, they merely assessed the mean value of the inclination angle of all cups implanted on bilateral hips. We suggest that the reason for this difference in surgical accuracy between the two groups may be due to immediate positive feedback from a vivid memory of the first-operated hip in a simultaneous procedure.

The influence of type of surgery on the functional outcome, quality of life, and health status in the present study appears to be significant. We demonstrated that the mean postoperative HHS, EQ-5D index, and EQ-VAS score in the simultaneous group were all significantly better than those of the staged group. Our findings do not agree with previous studies that have reported similar HHS postoperatively in the groups [2, 9, 25]. The reasons for this are unclear, but this could be caused by more accurate surgical procedures, no delayed rehabilitation time for the first-operated hip and reduced time lost from work in a simultaneous procedure.

With a mean follow-up of more than 60 months, we found the staged procedure resulted in an increase in postoperative prosthetic-related complications and revision surgery, especially for the second-operated hip compared to the simultaneous procedure. The reported arthroplasty-related complication rates and revision rates between the two procedures appear to vary [9, 25–27]. Berend et al. [2] reported a higher revision rate (3.9% vs. 0.5%; *P* = 0.010) and a higher dislocation rate in the simultaneous group. However, Rasouli et al. [8] demonstrated that local complications occurred significantly more frequently in a staged BTHA compared to a simultaneous BTHA (4.05% vs. 6.20%; *P* < 0.001). A possible explanation for the result in our study could be the superiority of surgical accuracy in simultaneous BTHA over staged BTHA.

The study has several limitations. First, this was a retrospective cohort study. Second, the study population was relatively small. A higher number would be more appropriate to generalize our findings. Third, preoperative clinical scores in regard to functional outcome and quality of life were not evaluated. Thus, perioperative improvements in each score could not be addressed. Fourth, we did not examine hip diseases other than ONFH, and the results of the current study may not be applicable to patients with other conditions such as primary or secondary osteoarthritis. The above limitations are offset by the strengths of the study, which was an analysis of a single-surgeon case series with the same approach. Accordingly, a potential bias related to the surgical technique may not be considered. In addition, in order to objectively assess the surgical accuracy, only ONFH was included in this study because, compared to other conditions, non-traumatic ONFH usually has relatively little radiographic evidence of acetabular abnormality and infrequent bilateral asymmetry of the acetabulum at the time of surgery [35].

## Conclusion

With greater surgical accuracy, better functional outcome, and higher quality of life, simultaneous BTHA has been shown to be superior to staged BTHA in patients with bilateral ONFH. This may be attributed to immediate feedback from a more recent surgery. Our results suggest that for medically operable patients, bilateral hip disease could be treated with a simultaneous procedure rather than a staged procedure to achieve a better surgical outcome.

## Abbreviations

ARCO: Association Research Circulation Osseous
ASA: American Society of Anesthesiologists
BMI: Body mass index
BTHA: Bilateral total hip arthroplasty
EQ-5D: EuroQol-5 dimension
EQ-VAS: EuroQol-visual analogue scale
HHS: Harris hip score
HLD: Head-to-lesser-trochanter distance
LLD: Leg length discrepancy
ONFH: Osteonecrosis of the femoral head
PACS: Picture archiving and communication system
SD: Standard deviation
THA: Total hip arthroplasty
